# Supplementary material for: Unraveling the mechanisms of PAMless DNA interrogation by SpRY-Cas9
Source: Nat Commun. 2024 Apr 30;15:3663. doi: 10.1038/s41467-024-47830-3 (PMC11061278; doi:10.1038/s41467-024-47830-3)
Supplement: Supplementary file 1 — Supplementary Information [file 41467_2024_47830_MOESM1_ESM.pdf]

Supplementary Information

Unraveling the mechanisms of PAMless DNA interrogation by SpRY-Cas9

Grace N. Hibshman, Jack P. K. Bravo, Matthew M. Hooper, Tyler L. Dangerfield, Hongshan Zhang, Ilya J. Finkelstein, Kenneth A. Johnson, David W. Taylor

Supplementary Figures

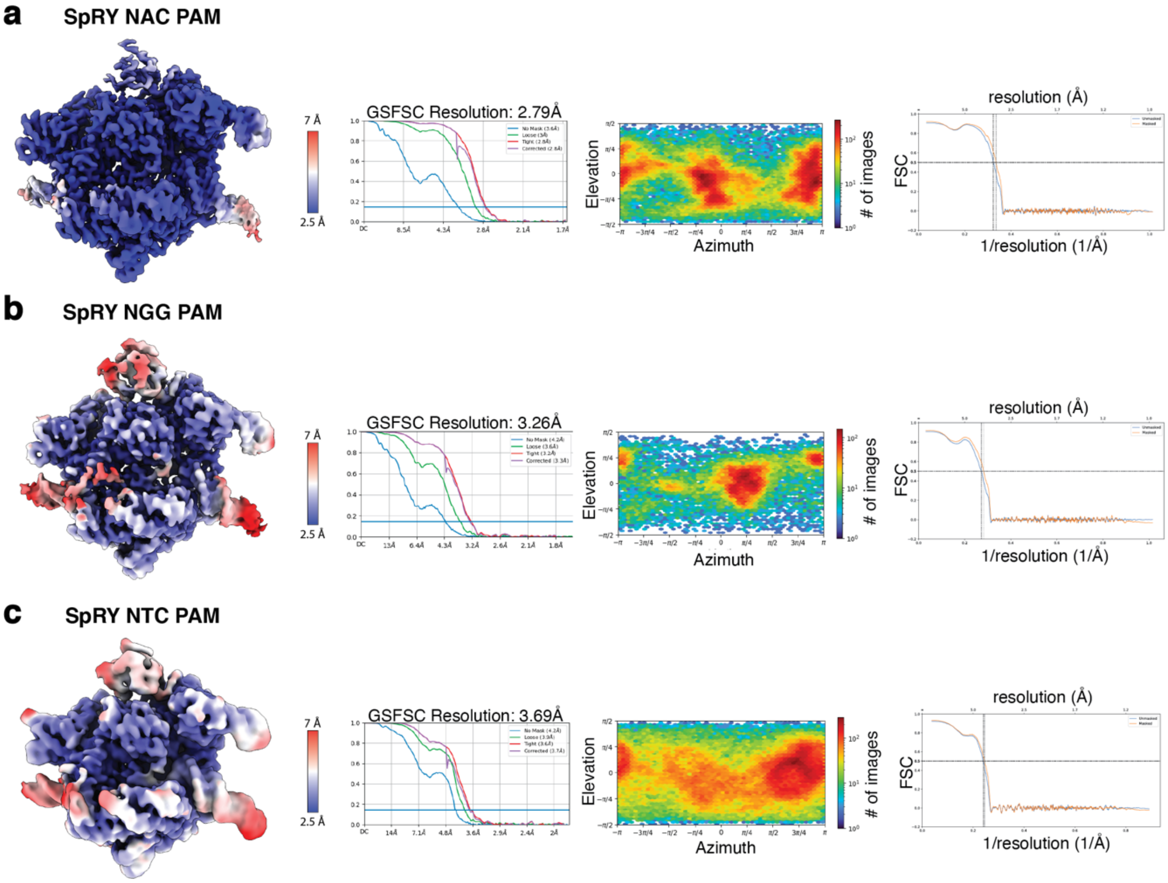

Supplementary Fig. 1. Cryo-EM data analysis of SpRY **a**, NAC PAM DNA **b**, NGG PAM DNA, and **c**, NTC PAM DNA.

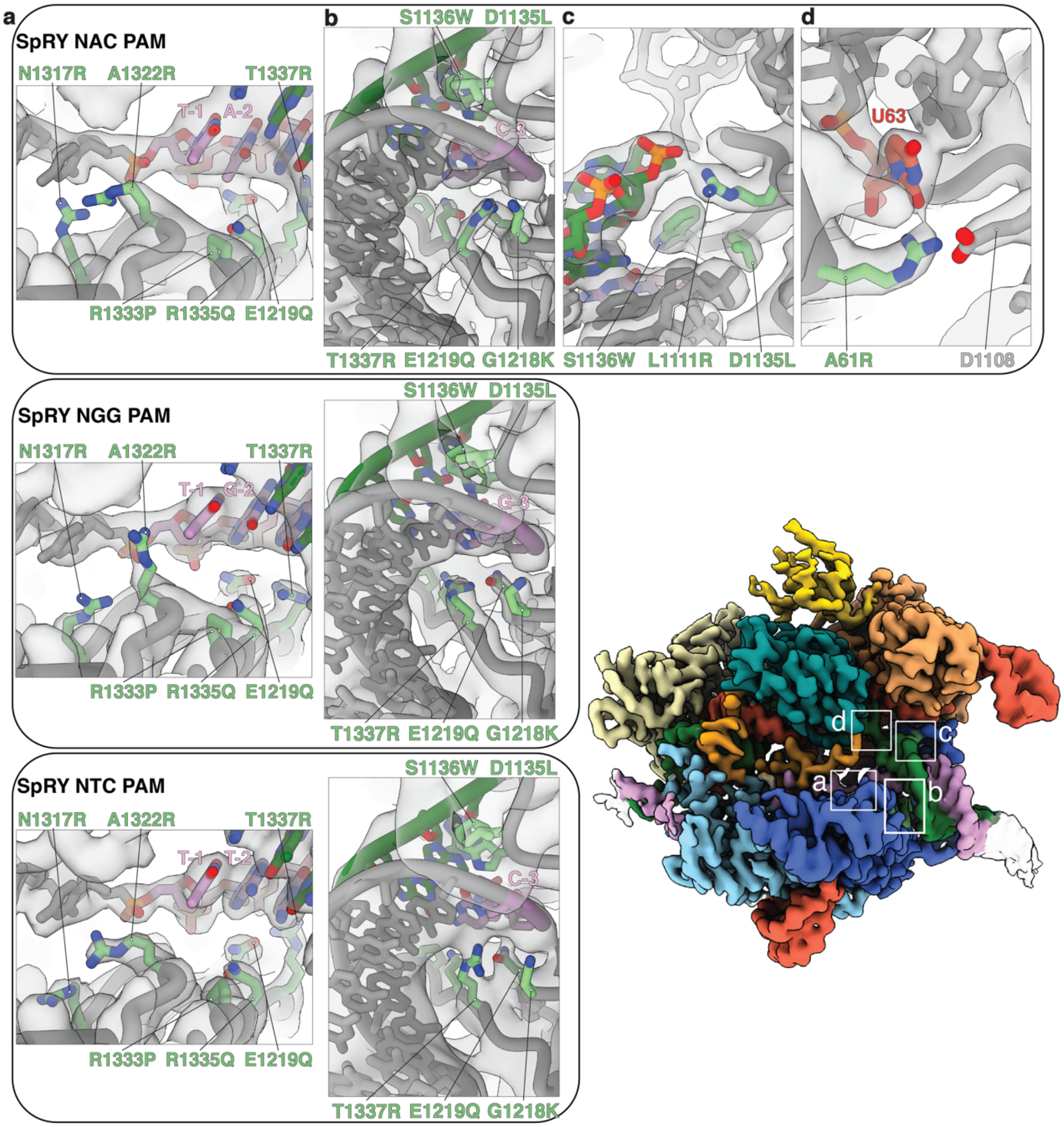

Supplementary Fig. 2. Detailed view of SpRY mutations and the rotamers adopted by different PAM sequences.

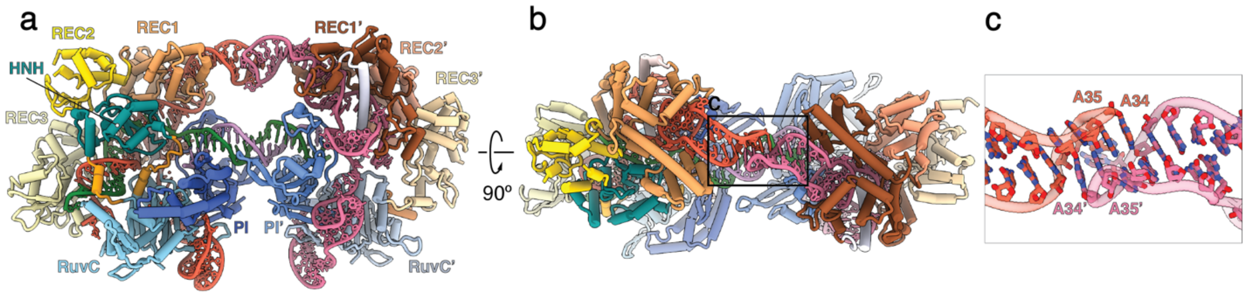

Supplementary Fig. 3. SpRYmer stem loop interaction. **a**, Overview of the SpRYmer atomic model with domains labeled. **b**, Top-down view of the SpRYmer atomic model. **c**, Detailed view of the stem loop kissing interaction.

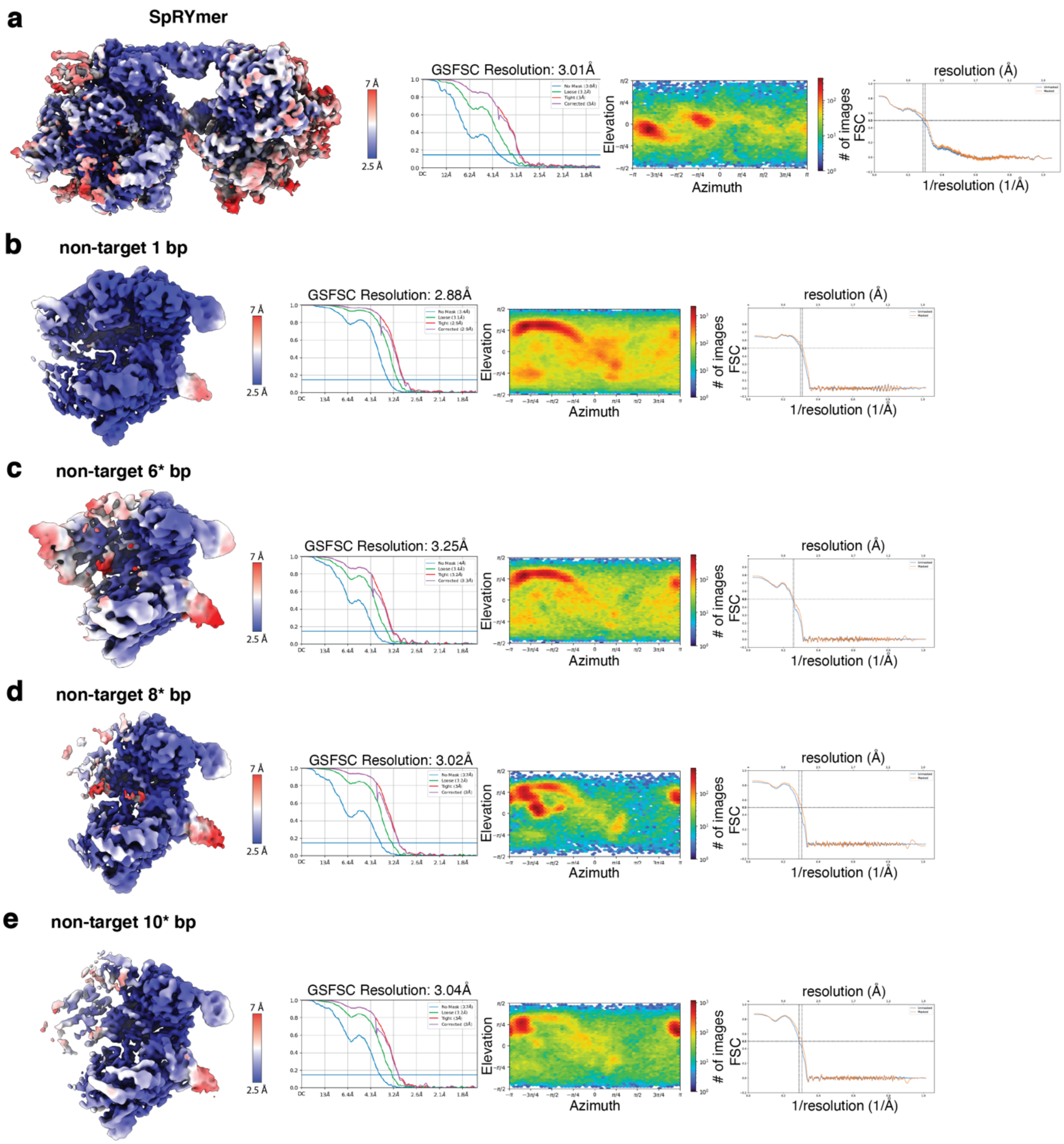

Supplementary Fig. 4. Cryo-EM data analysis of the **a**, SpRYmer **b**, SpRY bound to off-target DNA with 1 bp **c**, 6 bp **d**, 8 bp, and **e**, 10 bp of R-loop formed. *These structures contain mismatches.

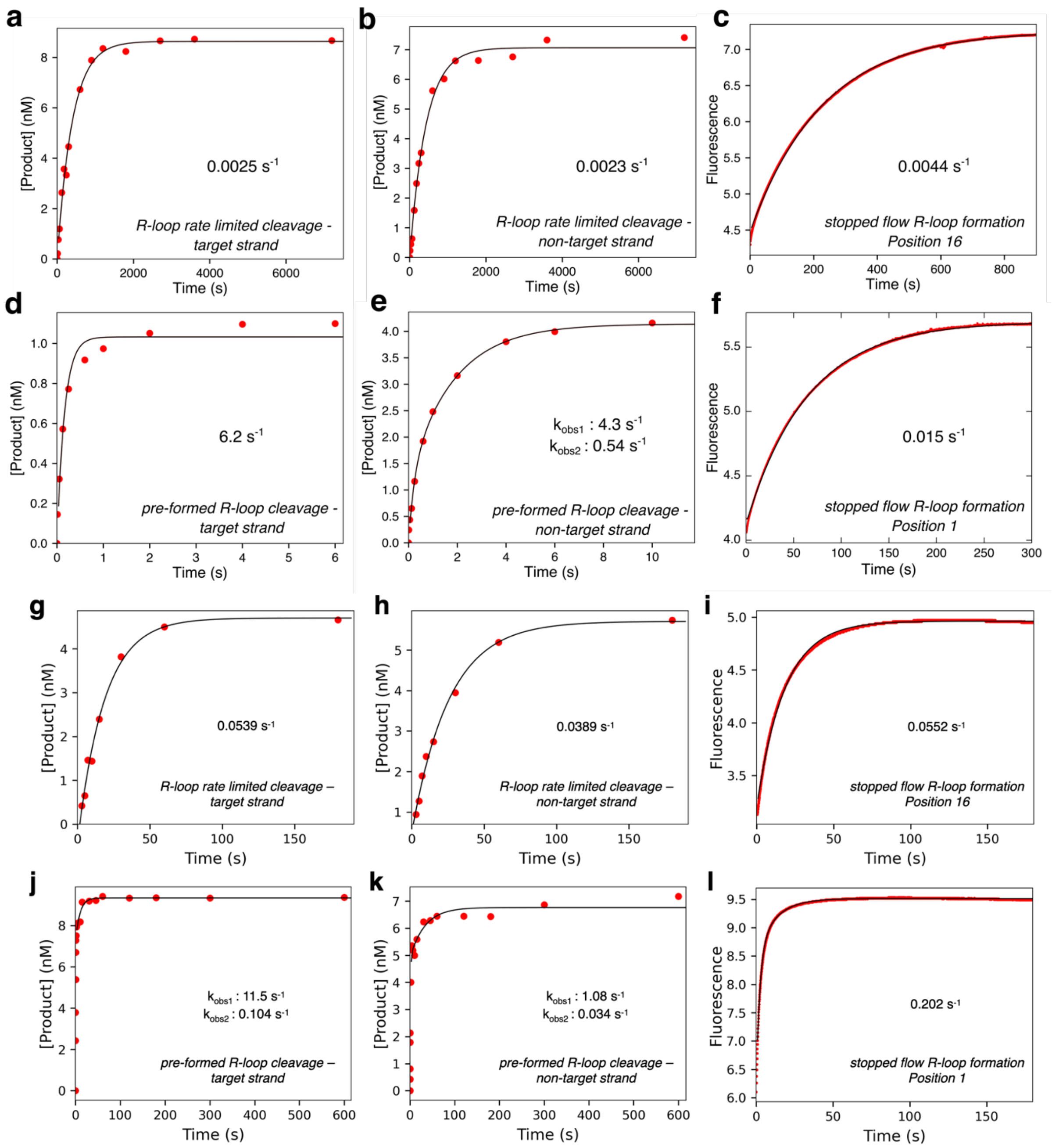

Supplementary Fig. 5. NGG PAM DNA cleavage kinetics for **a-f**, SpRY, and **g-l**, SpG.

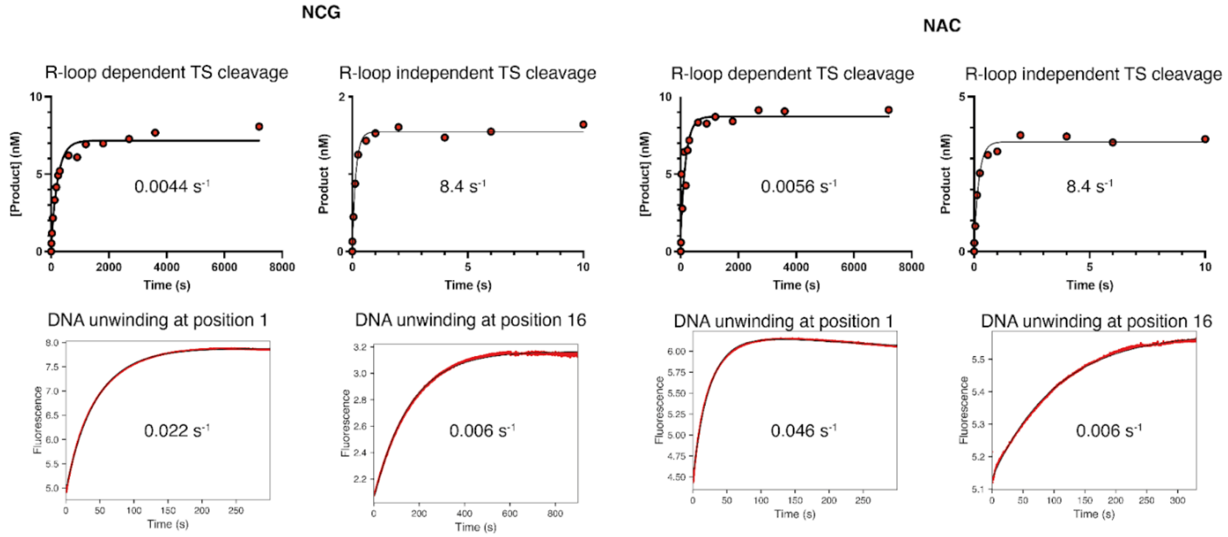

Supplementary Fig. 6. DNA cleavage and unwinding kinetics for NGC and NAC PAM substrates. The low amplitude of product for the Mg^{2+}-initiated reactions may be due to association of SpRY at partially complementary, off-target sites, thus reducing the effective enzyme concentration.

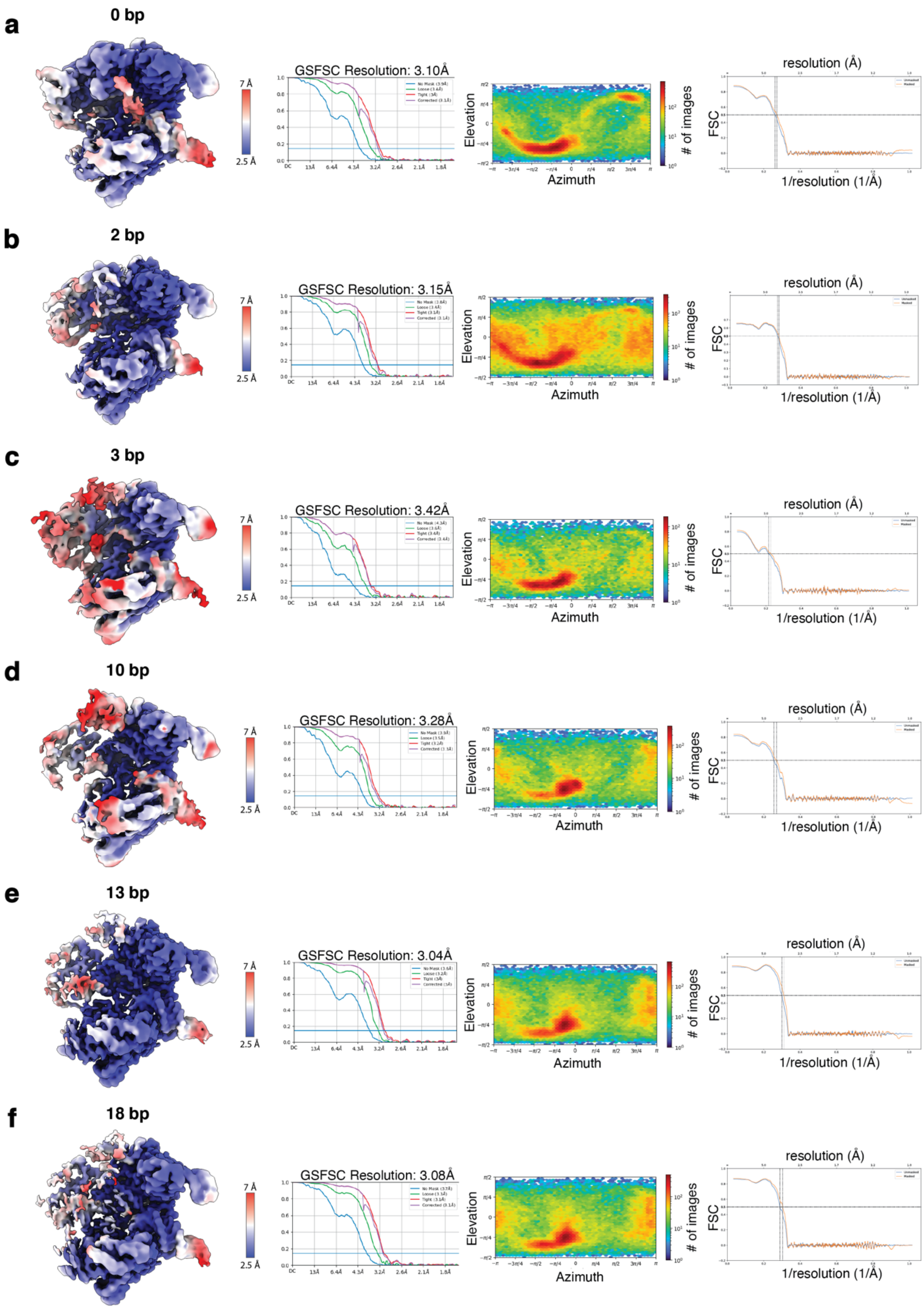
a
0 bp
7 Å
2.5 Å
GSFSC Resolution: 3.10Å
Elevation
Azimuth
# of images
resolution (Å)
FSC
1/resolution (1/Å)
b
2 bp
7 Å
2.5 Å
GSFSC Resolution: 3.15Å
Elevation
Azimuth
# of images
resolution (Å)
FSC
1/resolution (1/Å)
c
3 bp
7 Å
2.5 Å
GSFSC Resolution: 3.42Å
Elevation
Azimuth
# of images
resolution (Å)
FSC
1/resolution (1/Å)
d
10 bp
7 Å
2.5 Å
GSFSC Resolution: 3.28Å
Elevation
Azimuth
# of images
resolution (Å)
FSC
1/resolution (1/Å)
e
13 bp
7 Å
2.5 Å
GSFSC Resolution: 3.04Å
Elevation
Azimuth
# of images
resolution (Å)
FSC
1/resolution (1/Å)
f
18 bp
7 Å
2.5 Å
GSFSC Resolution: 3.08Å
Elevation
Azimuth
# of images
resolution (Å)
FSC
1/resolution (1/Å)

Supplementary Fig. 7. Cryo-EM data analysis of SpRY **a**, 0 bp **b**, 2 bp **c**, 3 bp **d**, 10 bp **e**, 13 bp **f**, 18 bp, and **g**, 20 bp (product state) R-loop intermediates.

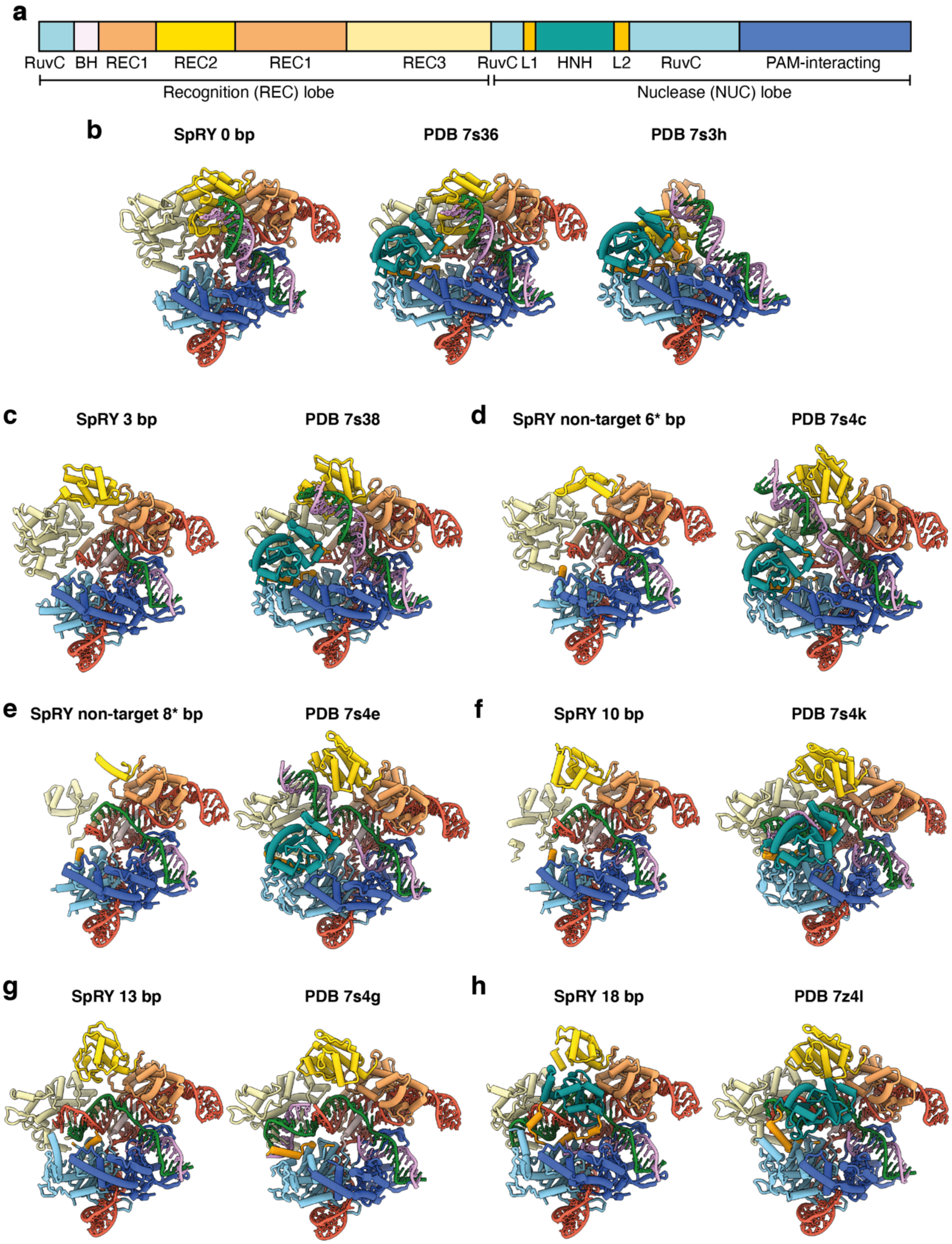

Supplementary Fig. 8. **a**, Comparison of SpRY 0 bp R-loop intermediate with previously determined Cas9 0 bp structures (PDB 7s36, 7s3h). **b**, Comparison of SpRY 3 bp R-loop intermediate with previously determined Cas9 3 bp structure (PDB 7s38). **c**, Comparison of SpRY 6* bp off-target DNA structure with previously determined Cas9 6 bp structure (PDB 7s4c). **d**, Comparison of SpRY 8* bp off-target DNA structure with previously determined Cas9 8 bp

structure (PDB 7s4e). **e**, Comparison of SpRY 10 bp R-loop intermediate with previously determined Cas9 10 bp structure (PDB 7s4k). **f**, Comparison of SpRY 13 bp R-loop intermediate with previously determined Cas9 12 bp structure (PDB 7s4g). **g**, Comparison of SpRY 18 bp R-loop intermediate with previously determined Cas9 18 bp structure (PDB 7z4l).

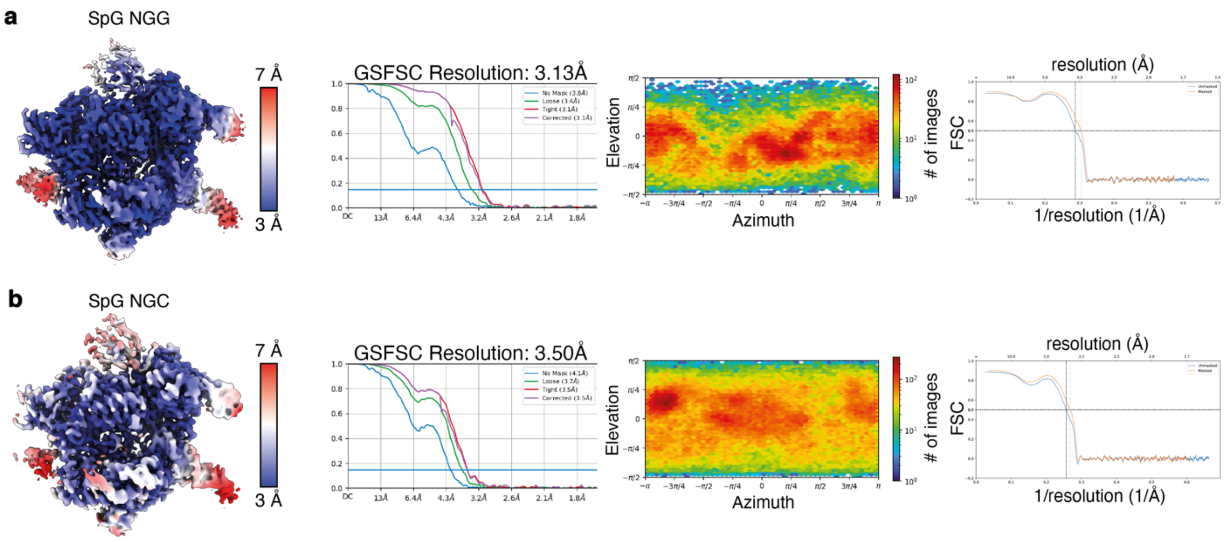

Supplementary Fig. 9. Cryo-EM data analysis of SpG bound to **a**, NGG PAM DNA, and **b**, NGC PAM DNA.

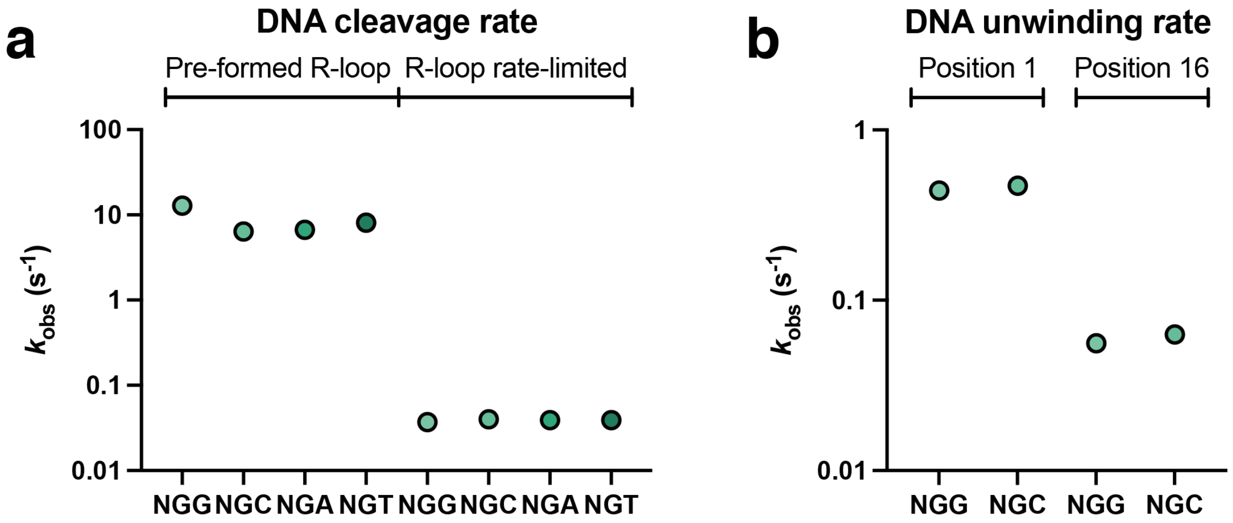

Supplementary Fig. 10. Observed rates for DNA cleavage and DNA unwinding for SpG on DNA substrates with various PAM sequences.

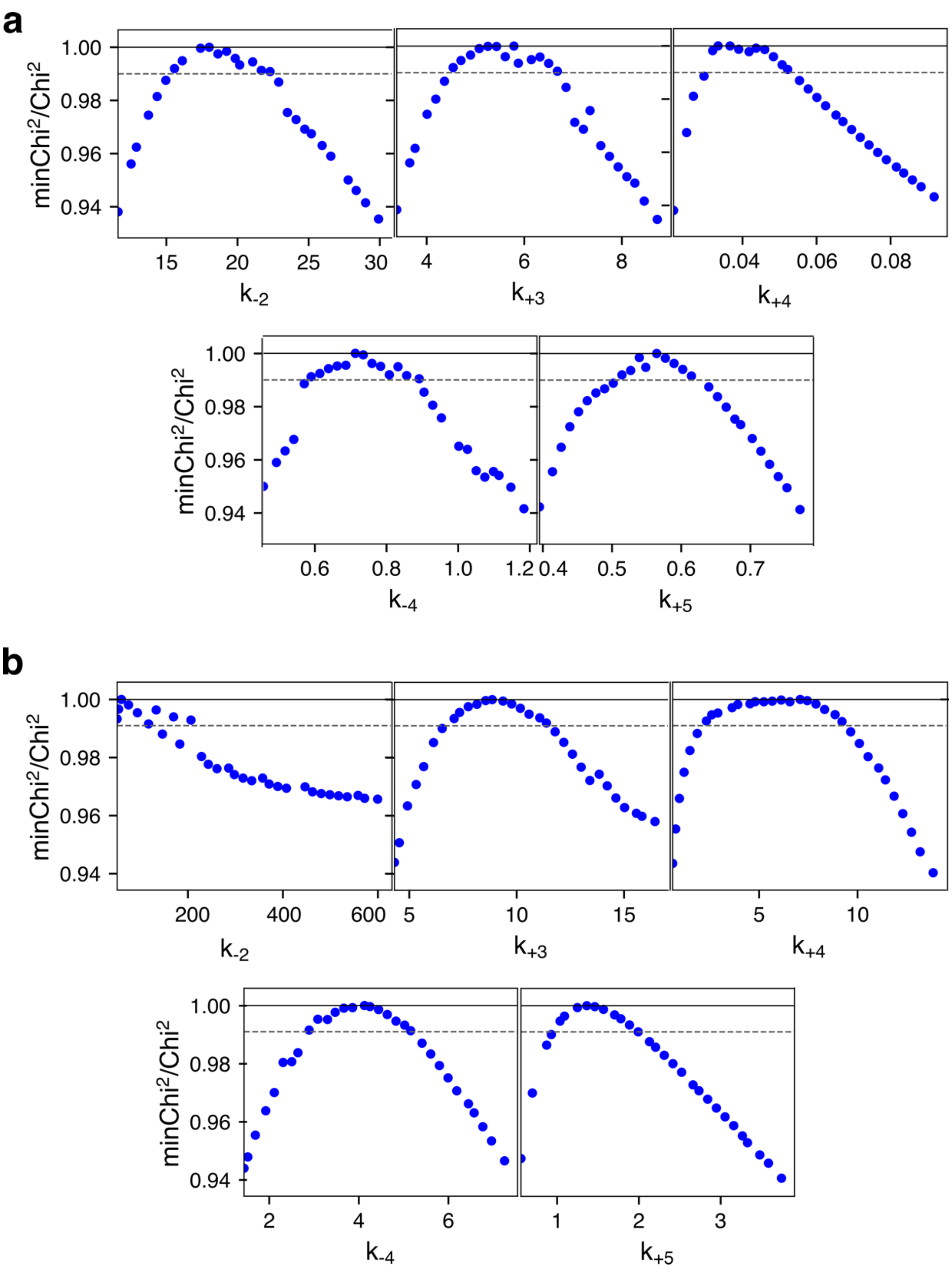

Supplementary Fig. 11. Confidence contours from global data fitting for **a**, SpRY, and **b**, SpG. Contours show the change in chi^2 as a function of values for individual rate. The dashed line indicates the chi^2 threshold corresponding to the 95% confidence interval used for reporting upper and lower limits on parameters listed in **Supplementary Table 3**.

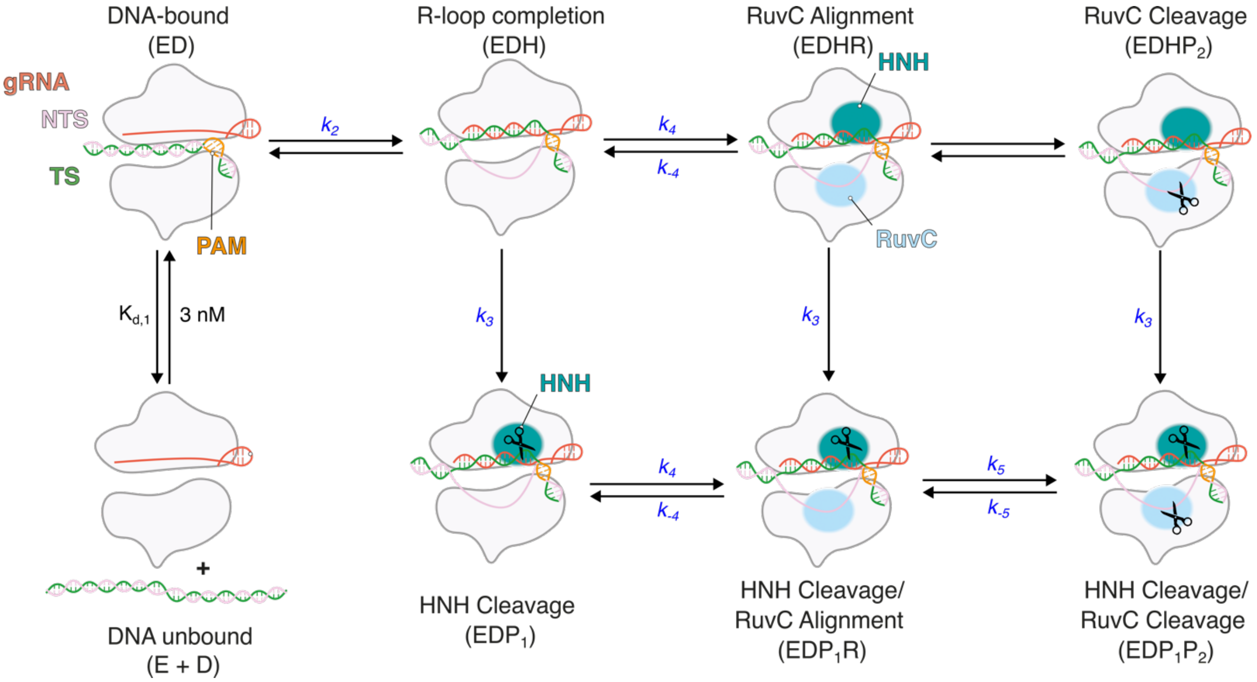

Supplementary Fig. 12. Full kinetic scheme used for global data fitting.

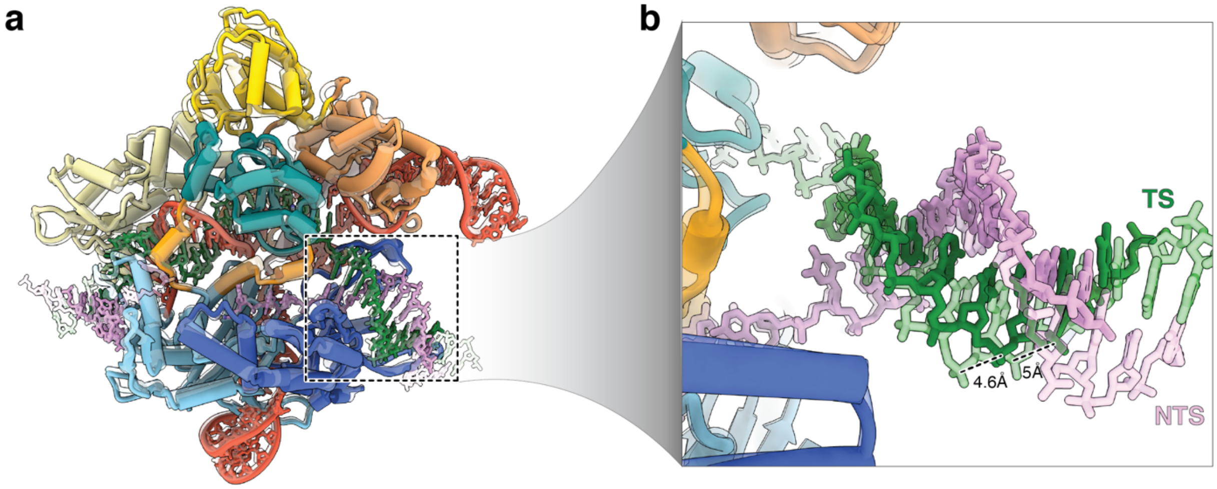

Supplementary Fig. 13. a, Overlay of SpRY bound to the NAC PAM DNA substrate and Cas9 (PDB 7s4x). **b**, Close up view of the PAM region of the structures shown in **a**. Cas9 is partially transparent, and SpRY is opaque.

Supplementary Tables

**Name**	**Sequence (5'-3')**	**Source**
TS NGG PAM	/6-FAM/agc tga cgt ttg tac tcc agc gtc tca tct tta tgc gtc agc aga gat ttc tgc t	IDT
NTS NGG PAM	agc aga aat ctc tgc tga cgc ata aag atg aga cgc TGG agt aca aac gtc agc t	IDT
TS NAC PAM	/6-FAM/agctgacgtttgtactGTagcgtctcatctttatgcgtcagcagagatttctgct	IDT
NTS NAC PAM	agcagaaatctctgctgacgcataaagatgagacgctACagtacaaacgtcagct	IDT
TS NTC PAM	/6-FAM/agctgacgtttgtactGAagcgtctcatctttatgcgtcagcagagatttctgct	IDT
NTS NTC PAM	agcagaaatctctgctgacgcataaagatgagacgctTCagtacaaacgtcagct	IDT
TS NGC PAM	/6-FAM/agctgacgtttgtactGCagcgtctcatctttatgcgtcagcagagatttctgct	IDT
NTS NGC PAM	agcagaaatctctgctgacgcataaagatgagacgctGCagtacaaacgtcagct	IDT
NTS NGG PAM Hot	/6-FAM/agc aga aat ctc tgc tga cgc ata aag atg aga cgc TGG agt aca aac gtc agc t	IDT
Scrambled TS	/56-FAM/agctgacgtttgtactccac*g*c*a*gagtagaaatacgcagagcagagatttctgct	IDT
Scrambled NTS	agcagaaatctctgctctgcgtatttct*a*c*t*c*t*g*cgtggagtacaaacgtcagct	IDT
TS NGG cold	agc tga cgt ttg tac tcc agc gtc tca tct tta tgc gtc agc aga gat ttc tgc t	IDT
TS NAC cold	agctgacgtttgtactGTagcgtctcatctttatgcgtcagcagagatttctgct	IDT
TS NGG cold	agctgacgtttgtactGAagcgtctcatctttatgcgtcagcagagatttctgct	IDT
NGG_NTS_tCo_16	agc aga aat ctc tgc tga cg[tCo] ata aag atg aga cgc tgg agt aca aac gtc agc t	Biosynthesis
NGC_NTS_tCo_16	agc aga aat ctc tgc tga cg[tCo] ata aag atg aga cgc tgc agt aca aac gtc agc t	Biosynthesis
NAC_NTS_tCo_16	agc aga aat ctc tgc tga cg[tCo] ata aag atg aga cgc tac agt aca aac gtc agc t	Biosynthesis
NGG_NTS_tCo_1	agc aga aat ctc tgc tga cgc ata aag atg aga cg[tCo] tgg agt aca aac gtc agc t	Biosynthesis
NGC_NTS_tCo_1	agc aga aat ctc tgc tga cgc ata aag atg aga cg[tCo] tgc agt aca aac gtc agc t	Biosynthesis
NAC_NTS_tCo_1	agc aga aat ctc tgc tga cgc ata aag atg aga cg[tCo] tac agt aca aac gtc agc t	Biosynthesis

Supplementary Table 1. List of DNA substrates used in this study. *Corresponds to phosphorotioate-modified DNA.

Supplementary Table 2. Cryo-EM data processing and modeling.

	SpRY NAC PAM 20 bp (EMD-40740) (PDB 8SRS)	SpRY NGG PAM (EMD-40681) (PDB 8SPQ)	SpRY NTC PAM (EMD-40705) (PDB 8SQH)	SpRY NAC PAM 0 bp (EMD-41073) (PDB 8T6O)	SpRY NAC PAM 2 bp (EMD-41074) (PDB 8T6P)	SpRY NAC PAM 3 bp (EMD-41085) (PDB 8T76)	SpRY NAC PAM 10 bp (EMD-41079) (PDB 8T6S)	SpRY NAC PAM 13 bp (EMD-41080) (PDB 8T6T)	SpRY NAC PAM 18 bp (EMD-41083) (PDB 8T6X)	SpRY non-target 1 bp (EMD-41084) (PDB 8T6Y)	SpRY non-target 6* bp (EMD-41086) (PDB 8T77)	SpRY non-target 8* bp (EMD-41087) (PDB 8T78)	SpRY non-target 10* bp (EMD-41088) (PDB 8T79)	SpRYmer (EMD-41093) (PDB 8T7S)
**Data collection and processing**														
Voltage (kV)	300 kV	300 kV	200 kV	300 kV	300 kV	300 kV	300 kV	300 kV	300 kV	300 kV	300 kV	300 kV	300 kV	300 kV
Electron exposure (e–/Å²)	80	80	49	80	80	80	80	80	80	80	80	80	80	80
Defocus range (µm)	-1.5 to -2.5													
Pixel size (Å)	0.8332	0.8332	0.94	0.8332	0.8332	0.8332	0.8332	0.8332	0.8332	0.8332	0.8332	0.8332	0.8332	0.8332
Symmetry imposed	C1													
Initial particle images (no.)	3,084,834	2,140,357	1,203,543	3,084,834	3,084,834	3,084,834	3,084,834	3,084,834	3,084,834	2,291,944	2,291,944	2,291,944	2,291,944	2,147,844
Final particle images (no.)	89,331	30,690	126,734	99,758	118,550	47,218	67,000	100,084	90,380	371,367	114,471	76,598	123,454	62,283
Map resolution (Å)	2.79	3.26	3.69	3.10	3.15	3.42	3.28	3.04	3.08	2.88	3.25	3.02	3.04	3.01
FSC threshold														
Map resolution range (Å)							2.5-7							
**Refinement**														
Initial model used (PDB code)	8SPQ	7S4X	8SPQ	7S36	7S38	8T6P	8SPQ	8T6X	8SPQ	8T6P	8T76	8T77	8T6S	8SRS, 4ZT0
Model resolution (Å)	3.0	3.5	4.1	3.7	3.6	4.0	3.7	3.2	3.3	3.2	3.8	3.3	3.3	3.3
FSC threshold	0.5	0.5	0.5	0.5	0.5	0.5	0.5	0.5	0.5	0.5	0.5	0.5	0.5	0.5
Map sharpening *B* factor (Å²)	67.4	67.1	156.8	90.4	102.2	84.1	88.9	91.0	97.8	109.3	97.6	82.4	90.6	49.0
Model composition	14151	14150	14110	11904	11174	11050	10405	12331	13445	11485	10752	9565	11942	24993
Non-hydrogen atoms	1341	1341	1341	1137	1108	1088	962	1168	1278	1148	1025	863	1146	2469
Protein	149	149	147	131	110	111	119	129	139	109	114	116	119	239
residues	4 Mg^{2+}, 1	4 Mg^{2+}	4 Mg^{2+}, 1	0	0	0	0	0	0	0	0	0	0	5 Mg^{2+}, 2
Nucleotides	H_2O		H_2O	0	0	0	0	0	3 Mg^{2+}	0	0	0	0	H_2O
Ligands														
R.m.s. deviations														
Bond lengths (Å)	0.005	0.005	0.006	0.0009	0.005	0.005	0.005	0.005	0.004	0.005	0.005	0.005	0.006	0.011
Bond angles (°)	0.85	0.944	1.043	1.141	0.892	1.021	0.994	1.212	0.722	0.983	0.976	0.979	1.281	1.946
Validation														
MolProbity score	1.35	1.35	1.64	1.23	1.33	1.42	1.46	1.45	1.29	1.28	1.42	1.39	1.54	1.44
Clashscore	3.86	4.82	7.09	4.59	4.64	5.81	6.38	5.90	4.44	4.83	6.21	5.40	6.89	4.75
Poor rotamers (%)	0.66	0.33	0.66	0.20	0.43	0.32	0.46	0.48	0.17	0.92	0.33	0.39	0.87	0.75
Ramachandran plot														
Favored (%)	97.00	97.52	96.32	98.23	97.54	97.49	97.45	97.32	97.70	97.89	97.62	97.52	97.08	96.81
Allowed (%)	3.00	2.48	3.68	1.68	2.46	2.51	2.55	2.68	2.30	2.11	2.38	2.48	2.92	3.19
Disallowed (%)	0.00	0.00	0.00	0.09	0.00	0.00	0.00	0.00	0.00	0.00	0.00	0.00	0.00	0.00

Parameter	Best fit		
	**SpRY**	**SpG**	**Cas9**
k_1	100 μM s^{-1} *	100 μM s^{-1} *	100 $\mu M^{-1}s^{-1}$*
k_{-1}	0.3 s^{-1} *	0.3 s^{-1} *	0.3 s^{-1} *
k_2	[> 0.02 s^{-1}]	[< 0.3 s^{-1}]	2.5
k_{-2}	16.1 s^{-1} (12.9, 20.1)	59.9 s^{-1} (44.2, 206)	1.2 s^{-1}
k_3	5.36 s^{-1} (4.29, 6.72)	8.9 s^{-1} (7.1, 11.4)	5.6 s^{-1}
k_4	0.038 s^{-1} (0.024, 0.048)	7.1 s^{-1} (2.3, 53.8)	1.8 s^{-1}
k_{-4}	0.65 s^{-1} (0.51, 0.77)	4.1 s^{-1} (2.8, 5.2)	2.3 s^{-1}
k_5	0.55 s^{-1} (0.48, 0.63)	1.36 s^{-1} (0.86, 2.0)	4.4 s^{-1}
k_{-5}	{ 0.0004 s^{-1} }	{ 0.0004 s^{-1} }	0.0004 s^{-1}

Supplementary Table 3. Rate constants derived from fitting the data in **Fig. 4c-g** and **Fig 6e-i** to the model shown i**n Supplementary Figure 12** are summarized. Values for SpRY and SpG in parentheses are lower and upper limits based on a 95% confidence interval from the confidence contour analysis. For SpRY and SpG, since k_2 was not well defined, it was locked at a lower limit of 0.02 s^{-1} and an upper limit of 0.3 s^{-1} for SpRY and SpG, respectively, for confidence contour calculations (locked rate constants are shown in brackets). Rates for Cas9 are taken from (26). The * indicates that rates for DNA binding and dissociation were not defined by the data but were locked at these rate constants to give a K_d of 3 nM. Values for k_{-5} in curly brackets were set at small values that did not change the results of the fitting to satisfy thermodynamic constraints and to allow generation of a free energy profile (20).
