# Supplementary material for: Unraveling the mechanisms of PAMless DNA interrogation by SpRY-Cas9
Source: Nat Commun. 2024 Apr 30;15:3663. doi: 10.1038/s41467-024-47830-3 (PMC11061278; doi:10.1038/s41467-024-47830-3)
Supplement: Supplementary file 2 — Reporting Summary [file 41467_2024_47830_MOESM2_ESM.pdf]

nature portfolio

Corresponding author(s): David W. Taylor

Last updated by author(s): 04/04/2024

Reporting Summary

Nature Portfolio wishes to improve the reproducibility of the work that we publish. This form provides structure for consistency and transparency in reporting. For further information on Nature Portfolio policies, see our Editorial Policies and the Editorial Policy Checklist.

Statistics

For all statistical analyses, confirm that the following items are present in the figure legend, table legend, main text, or Methods section.

n/a	Confirmed	
☐	☒	The exact sample size (n) for each experimental group/condition, given as a discrete number and unit of measurement
☐	☒	A statement on whether measurements were taken from distinct samples or whether the same sample was measured repeatedly
☒	☐	The statistical test(s) used AND whether they are one- or two-sided<br>*Only common tests should be described solely by name; describe more complex techniques in the Methods section.*
☒	☐	A description of all covariates tested
☒	☐	A description of any assumptions or corrections, such as tests of normality and adjustment for multiple comparisons
☒	☐	A full description of the statistical parameters including central tendency (e.g. means) or other basic estimates (e.g. regression coefficient) AND variation (e.g. standard deviation) or associated estimates of uncertainty (e.g. confidence intervals)
☒	☐	For null hypothesis testing, the test statistic (e.g. F, t, r) with confidence intervals, effect sizes, degrees of freedom and P value noted<br>*Give P values as exact values whenever suitable.*
☒	☐	For Bayesian analysis, information on the choice of priors and Markov chain Monte Carlo settings
☒	☐	For hierarchical and complex designs, identification of the appropriate level for tests and full reporting of outcomes
☒	☐	Estimates of effect sizes (e.g. Cohen's d, Pearson's r), indicating how they were calculated

Our web collection on statistics for biologists contains articles on many of the points above.

Software and code

Policy information about availability of computer code

Data collection	All datasets apart from NTC PAM were collected on an FEI Titan Krios cryo-electron microscope equipped with a K3 Summit direct electron detector (Gatan, Pleasanton, CA). Images were recorded with SerialEM v4.1 with a pixel size of 0.83 Å. Movies were recorded at 13.3 electrons/pixel/second for 6 s (80 frames) to give a total dose of 80 electrons/pixel. The NTC dataset was collected on a FEI Glacios cryo-TEM equipped with a Falcon 4 detector with a pixel size of 0.94 Å, and a total exposure time of 15s resulting in a total accumulated dose of 40 e/Å2 which was split into 60 EER fractions. All datasets were collected with a defocus range of -1.5 to -2.5 µm. Motion correction, CTF estimation and particle picking was performed on-the-fly using cryoSPARC Live v4.0.0-privatebeta.2. Further data processing was performed with cryoSPARC v.3.2. A total of 2,016 movies were collected for the NTC dataset, 4,293 movies for the NGG dataset, 3,546 movies for the NAC dataset, 10,151 movies for the R-loop intermediate dataset, and 8,765 movies for the off-target DNA dataset.
Data analysis	Data processing was performed with cryoSPARC v.3.2.NGG- and NGC PAM SpG complex datasets were processed as described for SpRY, with the addition of motion correction performed using MotionCor2 v 1.6.4. Nucleic acid alterations were made in Coot v1.1.07, and further modeling was performed using Isolde v1.6. The models were ultimately subjected to real-space refinement implemented in Phenix v1.21. All structural figures and videos were generated using ChimeraX v1.2. For single molecule studies, all images were exported as uncompressed TIFF stacks for further analysis in FIJI v2.15.1 (NIH) and MATLAB r2024a (The MathWorks).

For manuscripts utilizing custom algorithms or software that are central to the research but not yet described in published literature, software must be made available to editors and reviewers. We strongly encourage code deposition in a community repository (e.g. GitHub). See the Nature Portfolio guidelines for submitting code & software for further information.

Data

Policy information about availability of data

All manuscripts must include a data availability statement. This statement should provide the following information, where applicable:

- Accession codes, unique identifiers, or web links for publicly available datasets
- A description of any restrictions on data availability
- For clinical datasets or third party data, please ensure that the statement adheres to our policy

The structures and associated atomic coordinates have been deposited into the Electron Microscopy Data Bank (EMDB) and Protein Data Bank (PDB) with accession codes SpRY NAC PAM 20 bp (EMD-40740[https://www.ebi.ac.uk/emdb/EMD-40740] and PDB 8SRS[http://doi.org/10.2210/pdb8SRS/pdb].), SpRY NGG PAM (EMD-40681[https://www.ebi.ac.uk/emdb/EMD-40681] and PDB 8SPQ [http://doi.org/10.2210/pdb8SPQ/pdb]), SpRY NTC PAM (EMD-40705[https://www.ebi.ac.uk/emdb/EMD-40705] and PDB 8SQH[http://doi.org/10.2210/pdb8SQH/pdb]), SpRY NAC PAM 0 bp (EMD-41073[https://www.ebi.ac.uk/emdb/EMD-41073] and PDB 8T6O[http://doi.org/10.2210/pdb8T6O/pdb]), SpRY NAC PAM 2 bp (EMD-41074[https://www.ebi.ac.uk/emdb/EMD-41074] and PDB 8T6P [http://doi.org/10.2210/pdb8T6P/pdb]), SpRY NAC PAM 3 bp (EMD-41085[https://www.ebi.ac.uk/emdb/EMD-41085] and PDB 8T76[http://doi.org/10.2210/pdb8T76/pdb]), SpRY NAC PAM 10 bp (EMD- 41079[https://www.ebi.ac.uk/emdb/EMD-41079] and PDB 8T6S[http://doi.org/10.2210/pdb8T6S/pdb]), SpRY NAC PAM 13 bp (EMD- 41080[https://www.ebi.ac.uk/emdb/EMD-41080] and PDB 8T6T[http://doi.org/10.2210/pdb8T6T/pdb]), SpRY NAC PAM 18 bp (EMD- 41083 [https://www.ebi.ac.uk/emdb/EMD-41083] and PDB 8T6X[http://doi.org/10.2210/pdb8T6X/pdb]), SpRY off-target 1 bp (EMD- 41084[https://www.ebi.ac.uk/emdb/EMD-41084] and PDB 8T6Y[http://doi.org/10.2210/pdb8T6Y/pdb]), SpRY off-target 6* bp (EMD- 41086[https://www.ebi.ac.uk/emdb/EMD-41086] and PDB 8T77 [http://doi.org/10.2210/pdb8T77/pdb]), SpRY off-target 8* bp (EMD- 41087[https://www.ebi.ac.uk/emdb/EMD-41087] and PDB 8T78[http://doi.org/10.2210/pdb8T78/pdb]), SpRY off-target 10* bp (EMD- 41088[https://www.ebi.ac.uk/emdb/EMD-41088] and PDB 8T79 [http://doi.org/10.2210/pdb8T79/pdb]), SpRYmer (EMD-41093[https://www.ebi.ac.uk/emdb/EMD-41093] and PDB 8T7S[http://doi.org/10.2210/pdb8T7S/pdb]), SpG NGG PAM (EMD-41867[https://www.ebi.ac.uk/emdb/EMD-41867] and PDB 8U3Y[http://doi.org/10.2210/pdb8U3Y/pdb]), and SpG NGC PAM (EMD-41775[https://www.ebi.ac.uk/emdb/EMD-41775] and PDB 8TZZ[http://doi.org/10.2210/pdb8TZZ/pdb]). Source data are provided with this paper.

Research involving human participants, their data, or biological material

Policy information about studies with human participants or human data. See also policy information about sex, gender (identity/presentation), and sexual orientation and race, ethnicity and racism.

Reporting on sex and gender	NA
Reporting on race, ethnicity, or other socially relevant groupings	NA
Population characteristics	NA
Recruitment	NA
Ethics oversight	NA

Note that full information on the approval of the study protocol must also be provided in the manuscript.

Field-specific reporting

Please select the one below that is the best fit for your research. If you are not sure, read the appropriate sections before making your selection.

[x] Life sciences [ ] Behavioural & social sciences [ ] Ecological, evolutionary & environmental sciences

For a reference copy of the document with all sections, see nature.com/documents/nr-reporting-summary-flat.pdf

Life sciences study design

All studies must disclose on these points even when the disclosure is negative.

Sample size	No sample size calculation was performed. The number of particles needed was determined by the resolution of the structures required to support the claims of this study.
Data exclusions	No data was excluded.
Replication	Each experiment is representative of at least two independent experiments with successful replication.
Randomization	Particles are in randomized orientation at the beginning of data processing.
Blinding	No blinding was performed, as the data presented does not require blinding to avoid bias.

Reporting for specific materials, systems and methods

We require information from authors about some types of materials, experimental systems and methods used in many studies. Here, indicate whether each material, system or method listed is relevant to your study. If you are not sure if a list item applies to your research, read the appropriate section before selecting a response.

Materials & experimental systems

n/a	Involved in the study	
☐	☒	Antibodies
☒	☐	Eukaryotic cell lines
☒	☐	Palaeontology and archaeology
☒	☐	Animals and other organisms
☒	☐	Clinical data
☒	☐	Dual use research of concern
☒	☐	Plants

Methods

n/a	Involved in the study	
☒	☐	ChIP-seq
☒	☐	Flow cytometry
☒	☐	MRI-based neuroimaging

Antibodies

Antibodies used	Goat anti-rabbit polyclonal antibody (ICL Labs, #GGHL-15A) and digoxigenin monoclonal antibody (Life Technologies, #700772)
Validation	Control experiments with wild-type Cas9 confirmed proper labeling by the antibodies.

Plants

Seed stocks	NA
Novel plant genotypes	NA
Authentication	NA
